# Influence of age, frailty and non-cardiac comorbidities on survival in patients undergoing mitral valve edge-to-edge repair – a perspective from the German health care system

**DOI:** 10.1016/j.ijcha.2026.101990

**Published:** 2026-08-12

**Authors:** Kerstin Piayda, Nabor Keweloh, Bernhard Unsöld, Samuel Sossalla, Amin Polzin, Fabian Voss, Jafer Haschemi, Marcel Giemsa, Ursula Marschall, Beata Hennig, Michael Beil, Malte Kelm, Christian Jung

**Affiliations:** a Department of Cardiology and Vascular Medicine, University Hospital Giessen and Marburg and Medical Faculty Justus-Liebig-University, Giessen, Germany; b Department of Cardiology, Campus Kerckhoff of the Justus Liebig University Giessen, Bad Nauheim, Germany; c Department of Cardiology, Pulmonology and Vascular Medicine, University Hospital and Medical Faculty, Heinrich-Heine University, Duesseldorf, Germany; d Cardiovascular Research Institute (CARID), Medical Faculty, Heinrich-Heine University, Düsseldorf, Germany; e Department of Medicine and Health Services Research BARMER Statutory Health Insurance Fund Wuppertal, Germany.; f Department of Medical Intensive Care, Hadassah Medical Center and Faculty of Medicine, Hebrew University of Jerusalem, Jerusalem, Israel.; g Cardiovasular Research Institute Maastricht (CARIM), Faculty of Health, Medicine and Life Sciences, Maastricht, The Netherlands; h Cardiopulmonary Institute (CPI), Justus-Liebig-Universität Giessen, Giessen, Germany

**Keywords:** M-TEER, Mitral valve edge-to-edge-repair, Long-term survival, Clinical outcomes, Frailty, Comorbidities, Age

## Abstract

**Background and aims:**

The influence of age, frailty, and non-cardiac comorbidities on long-term survival in a large, real-world cohort of patients undergoing mitral valve edge-to-edge-repair (M-TEER) was investigated. In addition, associated healthcare resource utilization and expenditure were evaluated.

**Methods:**

Demographic data, data on frailty and co-morbidities were drawn from the anonymized database of the second largest sickness fund in Germany and analysed in regards of long-term survival.

**Results:**

Relevant data was available in 4896 patients. Very old patients (80–99 years) had an impaired survival as compared to younger ones (OR 1.2, 95% CI [1.07;1.49], *p* = 0.005). With increasing level of frailty (care level 1: OR 1.43, 95% CI [1.19;1.72], *p* = 0.0001; care level 2: OR 1.63, 95% CI [1.45;1.84], *p* < 0.0001; care level 3–5: OR 2.09, 95% CI [1.79;2.45], p < 0.0001), and increasing number of non-cardiac comorbidities (one: OR 1.55, 95% CI [1.32, 1.82], *p* < 0.001; two: OR 2.21, 95% CI [1.90;2.59], *p* < 0.001, three or more: OR 2.82, 95% CI [2.37;3.36], p < 0.001) survival was significantly impaired. Female sex seems to be protective (OR 0.75, 95% CI [0.68;0.82], *p* < 0.001), whereas right heart failure at baseline (OR 1.83, 95% CI [1.65;2.03], p < 0.001) has a negative effect on survival. Overall medical expenses 12-months before the procedure were equal to 12-months after M-TEER (12-months before: 5796€ IQR [856.5;14,856.0] vs. 12-months after: 4.184€ IQR [370;15,316].

**Conclusion:**

Age, frailty and comorbidities play a significant role in survival prediction of patients undergoing M-TEER. Overall medical expanses did not change after M-TEER however heart failure hospitalizations were less frequent.

## 1 Introduction

Mitral valve regurgitation (MR) is a common finding in the community setting and incidence increases with age. [1] Moderate to severe MR is associated with excess mortality and frequent heart failure hospitalizations if left untreated. [2] Mitral valve edge-to-edge repair (M-TEER) offers a clinical solution for patients who are deemed unfit for surgery [3], and current European guidelines on the management of valvular heart disease may consider M-TEER in selected patients with primary or secondary MR, after careful clinical evaluation and heart team discussion. [4] Recent randomized controlled trials like RESHAPE-HF2 [5] and MATTERHORN [6] further strengthen the role of M-TEER in daily clinical practice. Recent landmark randomized controlled trials, including RESHAPE-HF2 and MATTERHORN, were industry-sponsored trials. This contextualizes the independent, real-world nature of the present study, which draws on routine healthcare data from a large statutory health insurance fund without commercial funding or influence.

Already described predictors are the extend of MR reduction after M-TEER, moderate to severe tricuspid regurgitation at baseline and the presence of renal insufficiency and other comorbidities. [7], [8], [9], [10], [11], [12] Until now, no-large scale, real-world data exist, which allows us to estimate long-term mortality in patients undergoing M-TEER. Especially in this patient clientele, age, frailty and comorbidities play an evident role for patient selection and clinical decision making. Hence, we analysed data from a large German sickness fund with over eight million members, covering approximately 10 % of the German population to further elucidate on the influence of age, frailty and multimorbidity on long-term mortality after M-TEER.

## 2 Methods

Health insurance is compulsory in Germany and is either provided by statutory health insurance (covering around 89% of the population) or by substitutive private health insurance. Statutory health insurance is organized in over 90 sickness funds which act as third-party payers. [13] In addition to covering the cost for acute care, German residents are entitled to health insurance covering the cost for long-term care (either at home or at specialized institutions). The linked financial assistance increases with the extend of functional deficits (care level, so-called “Pflegegrad”). This creates incentives for the population to apply 1) if newly perceived functional impairments are present or 2) in case of functional status deterioration for re-assessment. Dedicated, medical assessors evaluate the autonomy and ability of each applicant in modules with different weightings. The overall score classifies the individual into five categories. This assessment results in a composite score which determines the grade of functional deficits (“Pflegegrad”) ranging from no significant impairment (grade 0), mild or moderate impairments (grades 1 and 2) to severe and very severe limitations (grades 3 and 4) with increased care requirements (grade 5). The grade is recorded by the BARMER health insurance organisation for all members in an anonymised database.

Although the Pflegegrad is a legally defined construct specific to the German long-term care system, it conceptually aligns with the WHO's internationally recognized framework of intrinsic capacity, encompassing domains such as locomotion, cognition, psychological well-being, and vitality. The care level can therefore be understood as an operationalized, legally binding proxy measure of intrinsic capacity within the German healthcare context. [14] A conceptual comparison between the Pflegegrad system and intrinsic capacity is displayed in Table 1.

**Table 1:** Classification of impairment of independence and abilities in the German health care system.

Aspect	Intrinsic Capacity (WHO)	Pflegegrad (Germany)
Perspective	Capacity-oriented	Impairment-oriented†
Primary aim	Functional health and healthy aging	Assessment of long-term care dependency
Multidimensional approach	Yes	Yes
Inclusion of cognitive and psychological domains	Yes	Yes (since 2017)‡
Legally binding	No	Yes
Grading		Care level 0: no significant impairments Care level 1: mild impairments Care level 2: moderate impairments Care level 3: severe limitations Care level 4: very severe limitations Care level 5: very severe limitations with increased care requirements

† The *Neues Begutachtungsassessment* measures degrees of independence, thereby indirectly capturing remaining functional capacity.

‡ Following the reform of the German Social Code Book XI (*Sozialgesetzbuch XI*, SGB XI) in 2017, cognitive and psychological impairments were formally integrated into the care dependency assessment, replacing the previous system based primarily on time-measured care needs.

Demographic data, data on frailty, co-morbidities and survival were drawn from the anonymized database of the second largest sickness fund in Germany (BARMER, www.barmer.de), which covers approximately 10 % of the German population. Additionally, the number of heart failure hospitalizations, doctor-patient contacts in the ambulatory setting and overall medical expenses 12 months before and after M-TEER were analysed.

**Statistics:** Continuous variables are presented as median with interquartile range (IQR) and categorical variables as absolute numbers and percentages. Group comparisons for continuous variables were performed using the Mann-Whitney *U* test, and chi-square or Fisher's exact test was applied for categorical variables, as appropriate. Long-term survival was assessed using Cox proportional hazards regression analysis, with results expressed as odds ratios (OR) with 95% confidence intervals (CI). Kaplan-Meier curves were generated to illustrate survival probabilities across subgroups. Prior to modelling, multicollinearity among all ordinal and categorical predictors with more than two levels was assessed using Spearman correlation coefficients. No strong correlations were identified between any variable pairs (all *r* < 0.2), confirming sufficient independence of model parameters. A two-sided *p*-value of <0.05 was considered statistically significant. All statistical analyses were performed using R (version 4.3.1R (version 4.3.1, www.r-project.org).

**Ethics:** This study is in line with the declaration of Helsinki and was approved by the local Ethics committee (No. 2024–2952).

## 3 Results

Demographic data, co-morbidities and long-term survival was available in 4896 patients. Information on frailty at index hospitalization for M-TEER and long-term survival was available in 4631 individuals, respectively. The baseline characteristics are shown in Table 2.

**Table 2:** Selected baseline characteristics divided by the level of impairment of independence and abilities.

	All patients	No impairments	Level 1	Level 2	Level 3–5
Number of. pts., n (%)	4896 (100)	3665 (74.8)	265 (5.4)	679 (13.8)	287 (5.8)
Age (Median, IQR)	81 (77–84)	81 (76–84)	83 (79–86)	82 (78–86)	82 (77–86)
Male (%)	2227 (45.5)	1777 (48.5)	81 (30.5)	249 (36.7)	120 (41.8)
Heart failure (%)	4739 (96.7)	3530 (96.3)	260 (98.1)	667 (98.2)	282 (98.2)
Left heart failure (%)	4624 (94.4)	3433 (93.6)	254 (95.8)	658 (96.9)	279 (97.2)
NYHA Class (Median, IQR)	4 (3–4)	3 (3–4)	4 (3–4)	4 (3–4)	4 (3–4)
Right heart failure (%)	2864 (58.5)	2001 (54.6)	174 (65.6)	473 (69.6)	216 (75.2)
Coronary artery disease (%)	4263 (87.1)	3170 (86.5)	235 (88.7)	604 (88.9)	254 (88.5)
Previous coronary artery bypass grafting (%)	790 (16.1)	596 (16.3)	41 (15.5)	109 (16)	44 (15.3)
Atrial fibrillation (%)	3965 (80.9)	2921 (79.7)	227 (85.6)	568 (83.6)	249 (86.7)
Arterial hypertension (%)	4744 (96.8)	3530 (96.3)	259 (97.7)	672 (97.5)	283 (98.6)
Peripheral artery disease	1034 (21.1)	698 (19)	58 (21.8)	181 (26.6)	97 (33.8)
Chronic pulmonary artery disease (%)	1451 (29.6)	975 (26.6)	102 (38.5)	256 (37.7)	118 (41.1)
Dementia (%)	254 (5.1)	139 (3.8)	17 (6.4)	50 (7.4)	48 (16.7)
Diabetes Type I (%)	257 (5.2)	172 (4.7)	17 (6.4)	44 (6.5)	24 (8.3)
Diabetes Type II (%)	2029 (41.4)	1438 (39.2)	115 (43.4)	326 (48.1)	150 (52.3)
Chronic renal failure (%)	3329 (67.9)	2375 (64.8)	188 (70.9)	524 (77.1)	242 (84.3)
Stage of chronic renal failure (Median, IQR)	3 (3–4)	3 (3–4)	3 (3–4)	3 (3–4)	3 (3–4)

### 3.1 Age

**Fig. 1:**
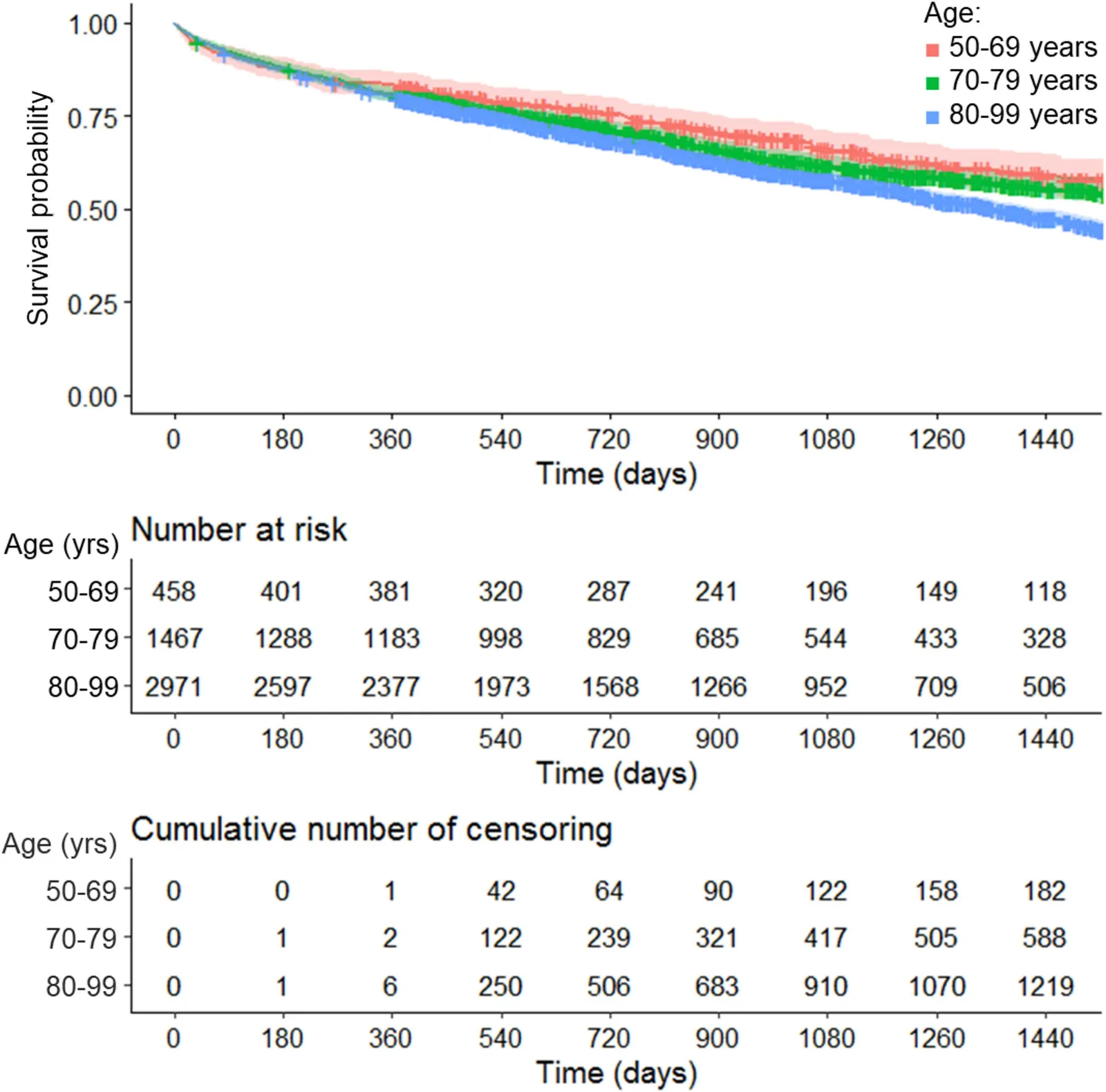
Long-term survival of M-TEER patients, stratified by age. Red: 50–69 years (young patients); green: 70–79 years (old patients), and blue: 80–99 years (very old patients).

Patients were split into three age groups (group 1: 50–69 years (young patients), group 2: 70–79 years (old patients), and group 3: 80–99 years (very old patients)), and long-term survival was analysed, [10] respectively. Group 1 comprised of 458 (9.4%) patients, group 2 of 1467 (29.9%) patients, and group 3 contained the largest patient sample with 2971 (60.7%) individuals. During a four-year period, 2122 (43.3%) of 4896 patients died. As compared to young patients, very old patients had a decreased survival rate (OR 1.3, 95% CI [1.18;1.63], *p* < 0.0001), whereas no significant difference was observed between young and old patients (OR 1.13, 95% CI [0.95;1.34], *p* = 0.149). Findings are illustrated in a Cox-regression analysis (Fig. 1).

### 3.2 Frailty

**Fig. 2:**
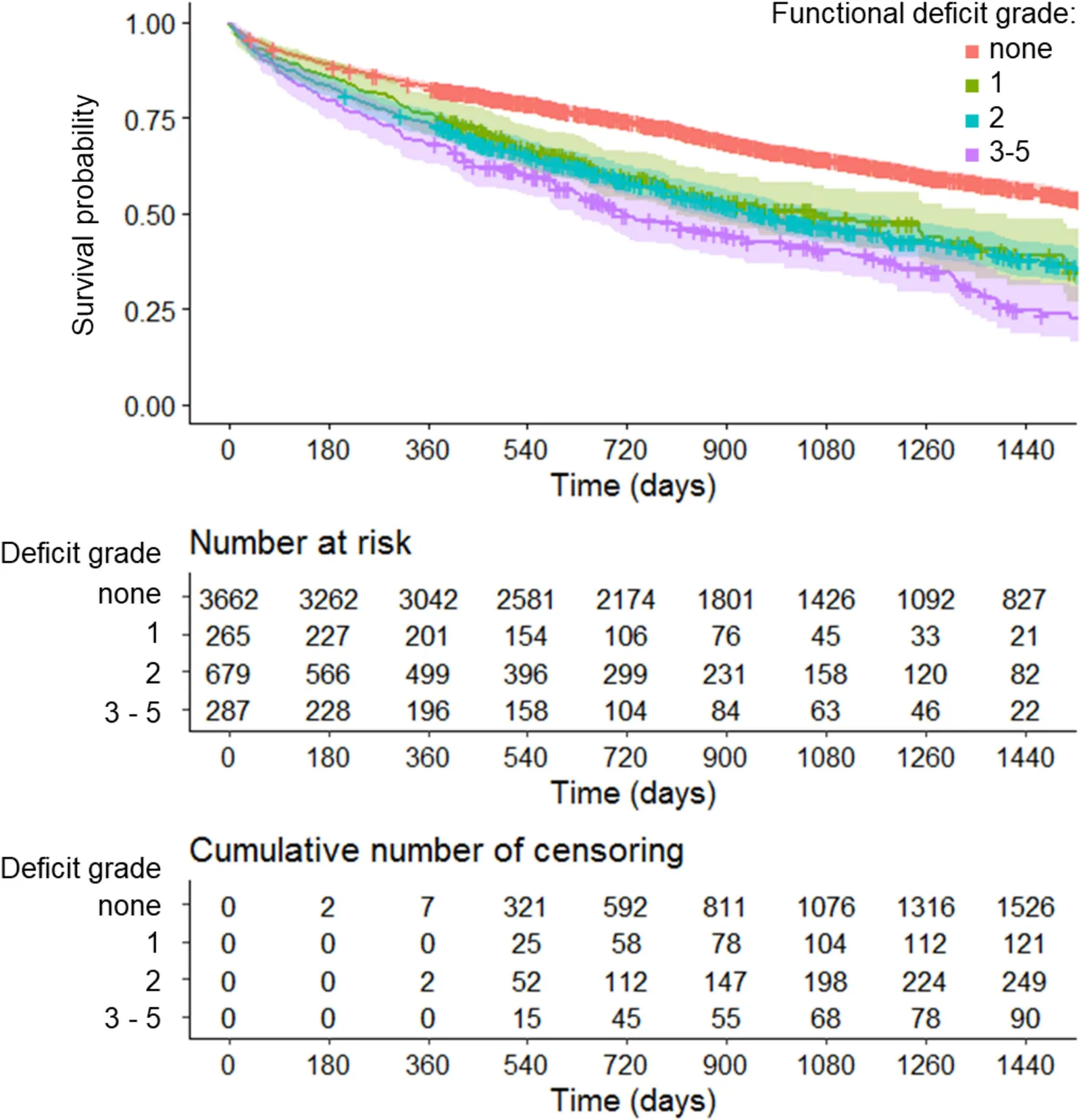
Long-term survival of M-TEER patients, stratified by impairments of independence and abilities. Red: individuals with no impairments of independence and abilities formed the largest group (care level 0); green: individuals with minor impairments of independence and abilities (care level 1); blue: individuals with significant impairments (care level 2); purple: individuals with heavy, severe, and severe impairments of independence and abilities with special requirements for nursing (care level 3–5).

Patients were stratified by the pre-described care levels. Individuals with no impairments of independence and abilities formed the largest group (care level 0, *n* = 3665, 79.1%). Two-hundred sixty-five (5.72%) patients had minor impairments of independence and abilities (care level 1), and *n* = 679 (14.7%) were assessed to have significant impairments (care level 2). For reasons of clarity, patients with heavy, severe, and severe impairments of independence and abilities with special requirements for nursing care were group together (*n* = 287, 6.2%, care levels 3–5). Baseline characteristics divided by the level of impairments of independence can be found in Table 2**.** With increasing level of care the survival probability was significantly reduced as compared to patients with no impairments of independence and abilities (care level 1: OR 1.46, 95% CI [1.21;1.75], *p* = 0.0001; care level 2: OR 1.49, 95% CI [1.32;1.67], *p* < 0.0001; care level 3–5: OR 1.75, 95% CI [1.49;2.05], p < <0.0001). The impact of impairments of independence and abilities on long-term survival is illustrated in Fig. 2**.**

### 3.3 Comorbidities and demographic aspects

**Fig. 3:**
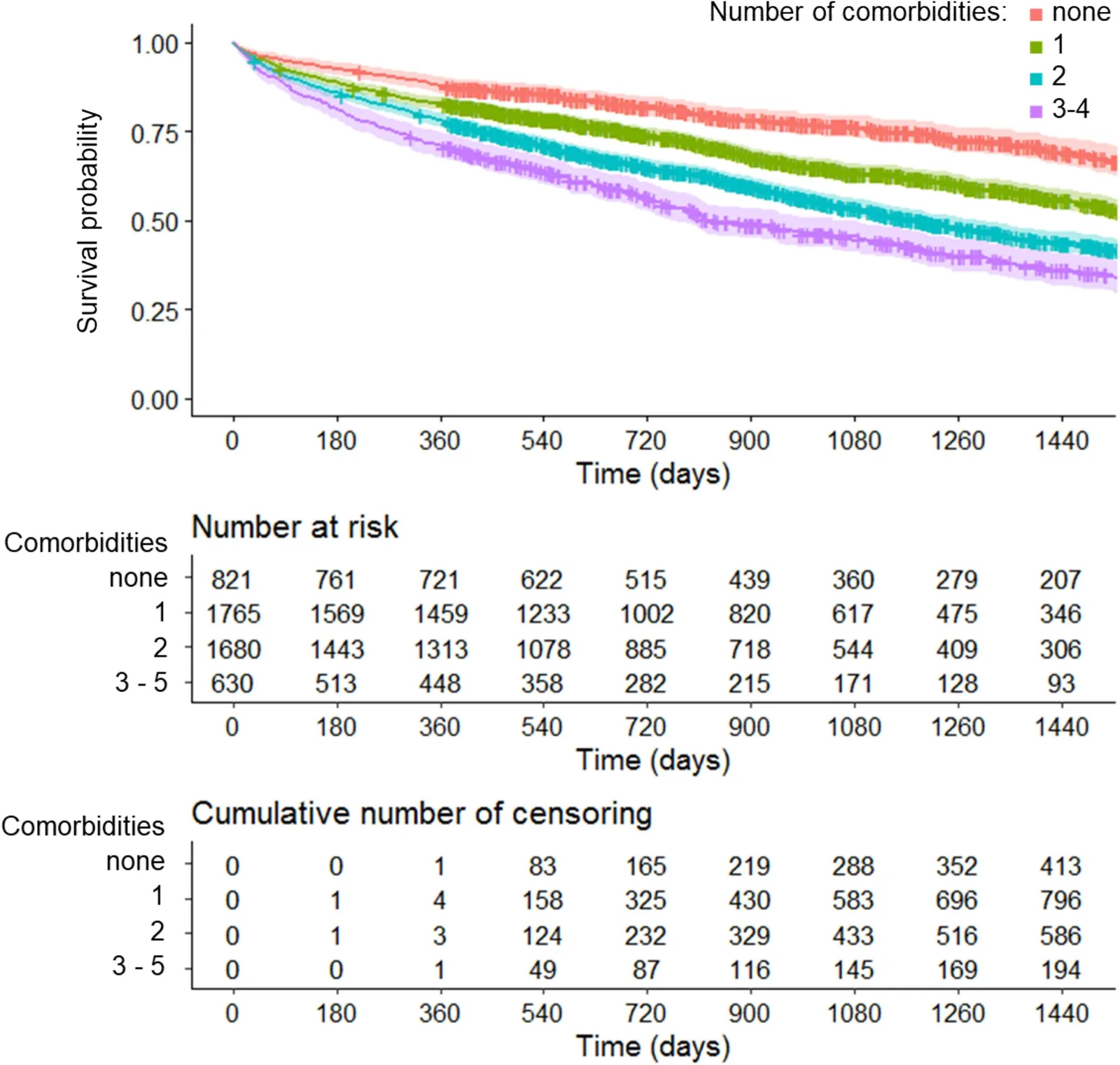
The influence on non-cardiac comorbidities on long-term survival of patients undergoing M-TEER. Non-cardiac comorbidities included chronic obstructive pulmonary disease, chronic kidney disease, dementia, Type two diabetes mellitus. Red: no non-cardiac comorbidity; green: one non-cardiac comorbidities; blue: two non-cardiac comorbidities; purple: three to four non-cardiac comorbidities.

Chronic pulmonary obstructive disease (COPD), chronic renal insufficiency, dementia and diabetes mellitus type II as the most important non-cardiac co-morbidities were analysed illustrating that long-term survival of patients undergoing M-TEER was influenced. A small share of patients did not suffer from any of the before mentioned comorbidities (*n* = 821, 16.7%). Most of the individuals presented with one non-cardiac comorbidity (*n* = 1765, 36.0%), followed by the group with two non-cardiac comorbidities (*n* = 1680, 34.3%). For reasons of clarity, patients with three or more non-cardiac comorbidities were pooled together (*n* = 630, 12.9%). Cox-regression analysis revealed that with increasing number of non-cardiac comorbidities survival probability decreases significantly (one non-cardiac comorbidity: OR 1.36, 95% CI [1.17, 1.59], *p* < 0.001; two non-cardiac comorbidities: OR 1.73, 95% CI [1.48;2.01], p < 0.001, three or more non-cardiac comorbidities: OR 2.04, 95% CI [1.71;2.43], p < 0.001). The influence of non-cardiac comorbidities on the survival of patients undergoing M-TEER is plotted in Fig. 3.

Female sex (OR 0.73, 95% CI [0.67;0.80], p < 0.001) and obesity (OR 0.89, 95% CI [0.81;0.98], *p* = 0.027) have a protective effect, whereas right heart failure at baseline (OR 1.83, 95% CI [1.65;2.03], p < 0.001) significantly impairs long-term survival. Other demographics which were evaluated to influence survival are displayed in Table 3.

**Table 3:** Baseline characteristics and impact on survival.

Variable	OR	95% CI	p-value
Age group: 70–79 years	1.13	0.95;1.34	0.149
Age group: 88–99 years	1.39	1.18;1.63	<0.0001
Care level 1	1.46	1.21;1.75	<0.0001
Care level 2	1.49	1.32;1.67	<0.0001
Care level 3–5	1.75	1.49;2.05	<0.0001
One non-cardiac comorbidity	1.36	1.17;1.59	<0.0001
Two non-cardiac comorbidities	1.73	1.48;2.01	<0.0001
Three and more non-cardiac comorbidities	2.04	1.71;2.43	<0.0001
Female sex	0.73	0.67;0.80	<0.0001
Number of clips	1.00	1.00;1.00	0.147
Right heart failure	1.74	1.58;1.92	<0.0001
Atrial fibrillation	1.08	0.96;1.22	0.173
Obesity	0.89	0.81;0.98	0.0274
Urban area	0.96	0.85;1.04	0.434

### 3.4 Health economic aspects

Overall medical expenses 12-months before the procedure were equal to 12-months after M-TEER (12-months before: 5796€ [856.5;14,856.0] vs. 12-months after: 4.184€ [370;15,316]). The number of doctor patient contacts in the ambulatory setting remained unchanged (12-months before: 19 [14;25] vs. 12-months after 17 [11;24], OR 0.99, 95% CI [0.98;1.00]). The number of heart failure hospitalizations (HFH) 12-months before M-TEER were higher as compared to 12-months after the procedure (12-months before: Mean 0.8 vs. 12-months after: 0.68; OR 1.14, 95% CI [1.06;1.23]). Findings, stratified by age group and level of impairment are listed in Table 4**.** Access to care was equally distributed between urban and rural areas (OR 0.94, 95% CI [0.85;1.04], *p* = 0.245) in Germany.

**Table 4:** Health care expenditure and heart failure hospitalization.

	Groups	12-months before M-TEER	12-months after M-TEER
Health care costs	50–69 years	7.206€ [1.102;19.408]	6.318€ [609,8; 23.627,2]
	70–79 years	5.859€ [917;15.187]	4.697€ [470;16.772]
	80–99 years	4.924,5€ [642.5;12.183]	3.536€ [358;12.764]
	No impairments	4.084€ [429;10.332]	3.463,5€ [356,5;13.163]
	Care level 1	7.692€ [2.885;15.736]	4.503€ [395;14.521]
	Care level 2	9.027€ [3.120;19.061]	5.578€ [611,5;18.789]
	Care level 3–5	13.218€ [4.973;25.607]	5.984€ [561;18.504]
Outpatient patient doctor contacts	50–69 years	17 [12;23]	18 [13;25]
	70–79 years	19 [13;25]	20 [14;26]
	80–99 years	19 [14;25]	18 [14;24]
	No impairments	18 [13;24]	19 [14;25]
	Care level 1	20 [14;26]	18 [14;25]
	Care level 2	20 [15;26]	19 [14;26]
	Care level 3–5	20 [15;27]	19 [14;25]

Care level 0: no significant impairments.

Care level 1: mild impairments.

Care level 2: moderate impairments.

Care level 3: severe limitations.

Care level 4: very severe limitations.

Care level 5: very severe limitations with increased care requirements.

## 4 Discussion

In contrast to industry-sponsored randomized controlled trials, the present study is entirely independent of commercial funding and free of sponsor-driven endpoint selection. Drawing on routine claims data from BARMER, covering approximately 10 % of the German population, our analysis reflects unselected, real-world clinical practice. This is of relevance, as it enables the inclusion of patient subgroups — such as the very elderly and those with significant frailty or multimorbidity — who are frequently underrepresented in sponsored trials yet constitute a substantial proportion of patients encountered in daily clinical practice. Our analysis of a large real-world patient cohort undergoing M-TEER shows comprehensively that age, frailty and comorbidities significantly influence long-term survival. These aspects are often interrelated and should be considered when it comes to patient selection.

### 4.1 Age

M-TEER plays a pivotal role in the treatment of patients with MR who have a prohibitive risk for surgery and has evolving clinical implications. Age alone is not necessarily a factor why patients are referred for transcatheter mitral valve repair, but comorbidities and frailty increase with age and are linked to adverse health care outcomes. In a primary care cohort of aging people, prevalence of multimorbidity was consistently high (89.3%) throughout all age groups, while frailty almost quadrupled (23.5% to 82.8%) from 65 to 99 years of age. [15]

Our real-world data sample shows that M-TEER is most used to treat very old (>80 years) and old (70–79 years) patients in Germany; and in very-old patients excess mortality is observed. Some randomized controlled trials [10], [16] and registry data [17] did not show that survival was significantly influenced by age. For example, in the COAPT trial, no two-year survival difference was found in-between the defined age groups (< 75 years, ≥ 75 years). However, in older patients HFH were reduced to a lesser extend as compared to younger patients. [16] On the other hand, in an analysis from the EuroSMR registry age, next to other criteria (i.e. renal failure, residual MR after M-TEER, NYHA class, left ventricular ejection fraction, and COAPT trial eligibility) age was an independent predictor for long-term survival. [12]

A relative survival analysis, as performed by the MitraSwiss registry investigators, may give more granular information in this context: the investigators showed that M-TEER in very-old patients with primary MR was able to restore the predicted life expectancy (described as relative survival, defined by the ratio between post M-TEER survival and expected survival in a matched age-, sex- and calendar period), whereas in patients with secondary MR life expectancy was bound to procedural success. [18] However, in daily clinical practice, M-TEER might be considered as a therapeutical option to primarily improve soft endpoints (i.e., amelioration of symptoms and reduction of HFH) rather than restoring relative survival, particularly in very old patients who might have already surpassed the mean predicted life-span of their age cohort.

### 4.2 Frailty

Frailty is a theoretical construct not linked to organ function but rather describes the age-related loss of haemostasis and resilience against stressors. [15], [19] It is considered to be one of the most problematic expression of population aging [20], although it lacks a uniform definition. Among individuals aged ≥75 years, frailty is present in 20–30% of individuals. [21] Hence, the majority of M-TEER patients is affected by frailty and several investigations elucidated on the role of frailty, although data on long-term outcomes remains scarce. An analysis from the Japanese multi-centre OCEAN trial used the clinical frailty scale [22] to show that all-cause mortality at 24-months is significantly linked to increased impairment of independence and abilities, and residual MR grade ≥ 2 after the intervention. [23] A variety of studies focuses on the association of frailty and short-term outcomes after M-TEER: Rios et al. [24] reviewed the national in-patient sample from the United States and could show that frailty was associated with increased in-hospital mortality, greater resource use, and incremental health care costs during the index hospitalization. A small-scale German study [25] (*n* = 213 patients, median follow-up: 1.17 years) showed that M-TEER can be performed with equal procedural success in frail and non-frail patients, and the procedure leads to short-term functional improvement.

Our data is in line with the current body of evidence, and is the first real-world, large patient sample showing that a gradual decrease in independence and abilities is linked to increased long-term mortality. The number of patients officially needing assistance due to impairments of independence and abilities seems rather small (25.15%).

It must be noted that in patients with very advanced age combined with significant functional deficits (care levels 3–5), the survival benefit of M-TEER may be attenuated, and that these findings should be considered in the heart team decision-making process. We emphasize that a comprehensive geriatric assessment — already reflected in the Pflegegrad system — should be an integral part of patient selection to avoid futile interventions, while also acknowledging the potential benefit in terms of symptom relief and quality of life, which is beyond the scope of the current dataset.

### 4.3 Comorbidities and demographic aspects

The high prevalence of multiple chronic conditions, defined as having two or more chronic diseases that last a year and require ongoing medical attention or limit activities of daily living, is the major driver for increased health care utilization in the elderly. [26], [27] In clinical practice, a certain overlap of frailty and multimorbidity exists: fewer multimorbid individuals also present with frailty, while most frail ones are also multimorbid. [28] Both, frailty and multimorbidity, are linked to poor health outcomes, increased mortality, and excessive health care costs. [29], [30], [31], [32] Multimorbidity has been investigated to a lesser extend in M-TEER patients. The Charlson comorbidity index is a widely used tool to estimate multimorbidity in patients and was able to predict mortality and clinical long-term outcomes. [33] The German Transcatheter Mitral Valve Intervention (TRAMI) registry (*n* = 722 patients, median follow-up time: 2.84 years) identified previous aortic valve implantation, prior cardiac decompensation, previous HFH, NYHA class IV, chronic kidney disease and a left ventricular ejection fraction below 30% as most predictive for long-term mortality. [17] Fewer studies investigate the influence of non-cardiac comorbidities, and mostly data on short-term survival after M-TEER is available. [34], [35]

In our analysis, female sex seems to be protective, however the current body of evidence is not conclusive: Agrawal et al. investigated that females have better adjusted long-term outcomes after M-TEER as compared to men. [36] In an analysis from the MitraSwiss registry both sexes had comparable 5-year outcomes, and M-TEER completely restored normal life expectancy in female patients with primary MR, which was not the case in women with secondary MR. Hence, further in-depth investigations are needed. [7] Additionally, obesity turned out to have a protective effect, although recent studies suggest that obesity-survival paradox does not exist and new anthropometric measures like the waist-to-height ratio may be more appropriate than the body mass index to predict adverse outcomes. [37]

Regarding cardiac co-morbidities, right heart failure at baseline proved to have a significant impact on long-term survival, which is in-line with several already published investigations. [38], [39], [40] Other well-known factors like ischemic cardiomyopathy, previous valve intervention, pulmonary artery pressure and tricuspid regurgitation [41] have not been further investigated in our patient sample.

### 4.4 Health economic aspects

With an aging population, health care systems are confronted with increasing health care demands. M-TEER has been proven to be cost-effective in different jurisdictions, such as the United Kingdom and as part of the COAPT trial in the United States of America. [42], [43] So far, no data is available for Germany. Our investigation could show that overall treatment costs for M-TEER patients is high and cannot be significantly reduced by transcatheter treatment of MR. Doctor patient contacts in the ambulatory setting remained unchanged, however HFH could be reduced, potentially leading to released pressure of hospitals facing diminished staff and financial resources.

With respect to clinical practice, we recommend that age, frailty (as assessed by structured tools such as the Pflegegrad or equivalent validated instruments), and the burden of non-cardiac comorbidities be systematically integrated into the heart team discussion prior to M-TEER. With respect to future investigations, we suggest that prospective studies incorporating quality-of-life endpoints alongside survival data are needed — particularly in the very elderly and frail — to more comprehensively inform the risk-benefit assessment of M-TEER in these populations.

## 5 Conclusion

Age, frailty and comorbidities play an important role for survival prediction in patients undergoing M-TEER. Very old patients, patients with impairment of independence and abilities, and an increasing number of non-cardiac comorbidities significantly dimmish long-term survival in this real-world patient sample of >4.500 individuals undergoing M-TEER in the German health care system. M-TEER effectively reduces the number of heart failure hospitalizations, even though overall medical expenses and number of doctor-patient contacts in the ambulatory setting remain unchanged 12-months after the intervention.

## 6 Limitations

The data lacks granularity in terms of procedural success and echocardiographic assessment. Health care costs and management are bound to the German health care system and findings may not be transferrable to other jurisdictions.

## CRediT authorship contribution statement

**Kerstin Piayda:** Writing – review & editing, Writing – original draft, Visualization, Supervision, Resources, Project administration, Methodology, Investigation, Conceptualization. **Nabor Keweloh:** Writing – review & editing, Resources, Project administration, Investigation. **Bernhard Unsöld:** Writing – review & editing, Supervision, Project administration, Methodology, Investigation. **Samuel Sosalla:** Writing – review & editing, Supervision, Resources. **Amin Polzin:** Writing – review & editing, Supervision, Project administration. **Fabian Voss:** Writing – review & editing, Resources, Investigation. **Jafer Haschemi:** Writing – review & editing, Resources, Investigation. **Marcel Giemsa:** Writing – review & editing, Software, Resources, Data curation, Conceptualization. **Ursula Marschall:** Writing – review & editing, Resources, Project administration, Funding acquisition, Formal analysis, Data curation, Conceptualization. **Beata Hennig:** Writing – review & editing, Validation, Software, Resources, Project administration, Investigation, Formal analysis, Data curation, Conceptualization. **Michael Beil:** Writing – review & editing, Visualization, Validation, Supervision, Methodology, Investigation, Formal analysis, Conceptualization. **Malte Kelm:** Writing – review & editing, Resources, Project administration. **Christian Jung:** Writing – review & editing, Writing – original draft, Validation, Supervision, Resources, Project administration, Methodology, Investigation, Funding acquisition, Formal analysis, Conceptualization.

## Disclosure statement

KP received speaker honoraria from Edwards Lifesciences and Abbott outside of this work.

All other authors have nothing to disclose.

## Declaration of competing interest

The authors declare that they have no known competing financial interests or personal relationships that could have appeared to influence the work reported in this paper.
